# HZE Radiation Non-Targeted Effects on the Microenvironment That Mediate Mammary Carcinogenesis

**DOI:** 10.3389/fonc.2016.00057

**Published:** 2016-03-11

**Authors:** Mary Helen Barcellos-Hoff, Jian-Hua Mao

**Affiliations:** ^1^Department of Radiation Oncology, University of California San Francisco, San Francisco, CA, USA; ^2^Lawrence Berkeley National Laboratory, Berkeley, CA, USA

**Keywords:** cosmic radiation, cancer risk models, ionizing radiation exposure, carcinogenesis process

## Abstract

Clear mechanistic understanding of the biological processes elicited by radiation that increase cancer risk can be used to inform prediction of health consequences of medical uses, such as radiotherapy, or occupational exposures, such as those of astronauts during deep space travel. Here, we review the current concepts of carcinogenesis as a multicellular process during which transformed cells escape normal tissue controls, including the immune system, and establish a tumor microenvironment. We discuss the contribution of two broad classes of radiation effects that may increase cancer: radiation targeted effects that occur as a result of direct energy deposition, e.g., DNA damage, and non-targeted effects (NTE) that result from changes in cell signaling, e.g., genomic instability. It is unknown whether the potentially greater carcinogenic effect of high *Z* and energy (HZE) particle radiation is a function of the relative contribution or extent of NTE or due to unique NTE. We addressed this problem using a radiation/genetic mammary chimera mouse model of breast cancer. Our experiments suggest that NTE promote more aggressive cancers, as evidenced by increased growth rate, transcriptomic signatures, and metastasis, and that HZE particle NTE are more effective than reference γ-radiation. Emerging evidence suggest that HZE irradiation dampens antitumor immunity. These studies raise concern that HZE radiation exposure not only increases the likelihood of developing cancer but also could promote progression to more aggressive cancer with a greater risk of mortality.

Epidemiological data on radiation therapy, occupational exposures, and accidental or terrorist radiological events have established the carcinogenic potential of sparsely ionizing radiation that includes γ-rays and X-rays. Less is known about the carcinogenic potential of densely ionizing radiation from accelerated particles recently implemented in the clinic and that are of a concern for space flight. The galactic cosmic radiation environment consists of high atomic number (*Z*) and energy (HZE) charged particles that are characterized by high linear energy transfer (LET) along the particle track, i.e., densely ionizing, in contrast to most terrestrial low LET radiations that are sparsely ionizing. The unique pattern of energy deposition incurred by HZE particle traversal is of often the primary focus in evaluating the biological effects of the galactic cosmic radiation on astronauts (1, 2). During a 3-year flight in extra-magnetospheric space, 3% of the cells of the human body would be traversed on average by one Fe ion (3). Cancer risk from exposure to the deep space radiation environment could constrain mission parameters for astronauts. The cancer incidence following radiotherapy is low but significant late tissue effect and, though the favorable dose distribution that reduces dose to normal tissue is thought to provide protection, that of HZE particle radiotherapy is yet unknown.

High *Z* and energy particle radiation is of particular concern for cancer because the limited experimental data to date indicate that the relative biological effect (RBE) for densely ionizing HZE particles is several-to-many fold greater than sparsely ionizing radiation. HZE particles have a high RBE for many biological end points (4); however, some HZE biological effects are not observed following sparsely ionizing radiation (5) and some radiation effects, such as genomic instability, do not show classic dose dependence (6). As a consequence, measurements of individual biological events and their dose dependence do not describe how an organism will respond to radiation damage. HZE particles traversing a cell nucleus cause difficult to repair clustered DNA damage that is classified as a radiation targeted effects (RTE), i.e., due to the deposition of energy in the cell. Radiation exposure also elicits complex changes in signaling and phenotype, which are called non-targeted effects (NTE) because they are often observed in the neighbors or daughters of irradiated cells.

Radiation is classified as a complete carcinogen in the etiology of human tumors, including breast cancer, lung cancer, lymphoma, liver carcinoma, sarcoma, and glioma (7). Radiation-induced DNA damage elicits a rapid and efficient repair network, but the occasional misrepair of these lesions results in mutations, translocations, deletions, and amplifications, which are also hallmarks of cancer cells. Many risk models use the frequency of these RTE as the basis for estimating cancer risk. Such models assume that the probability of cancer is proportional to DNA damage and, hence, exposure, which is consistent with epidemiological association of cancer risk and polymorphisms in certain genes in the DNA repair pathway (8).

The risk paradigm broadly based on RTE, that is direct DNA damage, has been challenged by at least two classes of NTE: first, the demonstration that descendants of irradiated cells exhibit non-clonal damage (i.e., radiation-induced genomic instability) or altered phenotype; second, the designation of so-called “bystander” radiation effects, in which non-irradiated cells respond to signaling by irradiated cells (6). NTE can be functionally defined by particular experimental strategies (e.g., bystander experiments and media transfer) and occur by various mechanisms that involve gap junctions, soluble factors, and phenotypic transition that differ between cell types and between *in vitro* and *in vivo* models.

The crucial question is to determine under what conditions and to what extent NTE contribute to human health risks. Recent experimental studies of radiation carcinogenesis following low- and high LET radiation exposures are concerned with how complex organismal responses to radiation interact across levels of organization and time scales to impede or promote malignant processes (9). Mechanistic understanding of cancer has become much more detailed over the last two decades. There is growing recognition that cancer as a disease results from a systemic failure, in which many cells other than those with oncogenic genomes determine the frequency of clinical cancer (10). The challenge to predicting health effects in irradiated humans is to understand how complex radiation responses culminate in pathology.

## Carcinogenesis in Context

The understanding of cancer as a result of systemic failure, in which many cells other than those with oncogenic mutations/alterations determine the frequency and characteristics of clinical cancer, underscores tissue dysfunction, in which cancer cells are highly intertwined with the microenvironment (11, 12). Both tissue and organismal biology are subverted during malignant progression (13). More than a quarter of a century ago, studies by Mintz and Pierce demonstrated that malignancy could be suppressed by contact with normal tissues (14, 15). Many have even argued that disruption of the cell interactions and tissue architecture can be the primary drivers of carcinogenesis (16–20). Recent experiments with engineered models have focused on identifying the type and means by which normal cells mediate the development of cancer (21–24), but it is clear that host cells, e.g., stromal cells and bone marrow-derived cells (BMDC), sculpt carcinogenesis in a complex process that can either eliminate or accelerate malignancy.

Recent studies demonstrate that host biology is altered even before cancer is evident. A systems biology approach by Hanash and colleagues characterized the plasma proteome response in the inducible HER2/neu mouse model of breast cancer during tumor induction, progression, and regression. Mass spectrometry data derived from approximately 1.6 million spectra identified protein networks associated with tumor development. Some networks were derived from the tumor microenvironment and some from tumor cell secreted or shed proteins. The observed alterations developed prior to cancer detection, increased progressively with tumor growth, and reverted toward baseline with tumor regression. Importantly, these findings were mirrored with findings resulting from in-depth profiling of circulating proteins using prediagnostic plasma samples from women who participated in the Women’s Health Initiative study and who subsequently developed breast cancer (25–27).

Although the prevailing radiation health paradigm focuses on radiation-induced DNA damage leading to mutations, numerous studies over the last 50 years have provided evidence that radiation carcinogenesis is more complex than generally appreciated [reviewed in Ref. (28)]. Terzaghi-Howe demonstrated that the expression of dysplasia *in vivo* and neoplastic transformation in culture of irradiated tracheal epithelial cells is inversely correlated to the number of cells seeded (29–32) and identified TGFβ as a key mediator (33). Our lab used a *Trp53* mutant mammary cell line to show that irradiating only the host increased the development of frank tumors fivefold (34). Saran and colleagues showed that partial body irradiation at a young age promotes *Ptch* mutant medulloblastoma (35).

**Figure 1:**
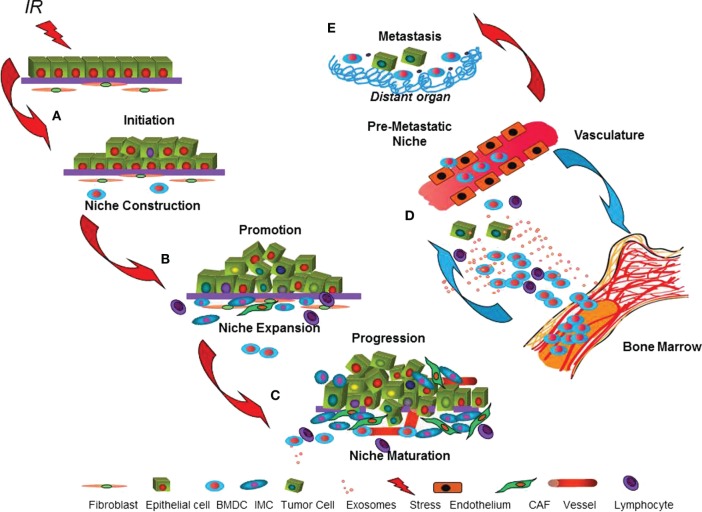
**The dynamic cancer niche**. The cartoon depicts parallel processes postulated to occur in the target epithelium and microenvironment during multistage epithelial carcinogenesis. **(A)** Misrepaired DNA damage caused by radiation can malignantly *initiate* epithelial cells. Radiation effects on cell signaling and phenotype may promote concomitant niche *construction* by local or systemically recruited cells that improve initiated cell survival. **(B)** Within the epithelium, *promotion* is considered to be acquisition of additional genetic aberrations or epigenetic traits that enable malignancy. In parallel, niche *expansion*, due to signals produced by either the initiated epithelium or by the niche cells that support them, conscripts stromal cells and bone marrow-derived cells (BMDC). **(C)** *Maturation* of the tumor microenvironment that enables angiogenesis, immune suppression, and invasion is necessary for tumor *progression*. **(D)** Systemic influences, including signaling to and from *vasculature* and *bone marrow*, contribute throughout multistage carcinogenesis *via* participation of BMDC, lymphocytes, and immature myeloid cells (IMC) and their secreted cytokines and exosomes. **(E)** Some cancers are able to initiate new microenvironments, the *pre-metastatic niche*, in distant organs that facilitate *metastasis*.

Many studies using oncogenic mouse models indicate that the stroma is highly involved in early malignancy (36), which supports the idea of reciprocal evolution of the malignant cell and the tumor microenvironment (10). Although it is clear that stroma composition and signaling is altered in human breast cancer (37), less is known about how and when stroma contributes to carcinogenesis and how carcinogens, such as radiation, might alter these processes. We postulate that the tumor microenvironment is built through rate-limiting steps of construction, expansion, and maturation that parallel initiation, promotion, and progression during multistage carcinogenesis (10). Construction of a “pre-cancer niche” is the necessary first step to generate a tumor microenvironment that is essential for initiated cells to survive and evolve into clinically evident cancers (Figure 1). The evolution of the tumor microenvironment *via* stromal cells and BMDC during subsequent niche expansion during promotion is mediated by cytokines secreted by either the initiated epithelial cells or those host cells recruited to the niche. Maturation of the tumor microenvironment, as evidenced by angiogenesis escape from immune suppression and generation of a stroma permissive for growth and often invasion, occurs during progression. Importantly, signaling is not just local but can also be mediated by cells, cytokines, and exosomes transported by the vasculature between the nascent cancer and distant sites include the bone marrow, which may reciprocate by expansion of cells, such as immature myeloid cells (IMC) that support tumor growth. Indeed, the pre-metastatic niche, first described by Lyden and colleagues, pre-dates and facilitates metastatic disease (38).

This model postulates that cancer survival and proliferation is as much a function of the successful niche construction as it is of specific cancer cell mutations. Indeed selective pressure for neoplastic mutations may be imposed by the composition of the niche, as well as by immune editing (39). Consequently, cancer represents an emergent property that requires a comprehensive analysis of the cell–cell interactions in the entire niche. Moreover, in contrast to initiation, which is a stochastic process by nature, niche construction represents a robust target for native immunosuppression and a potent target for cancer prevention. If microenvironments induced by radiation can promote neoplastic progression in unirradiated epithelial cells, events outside of the (targeted) box may significantly increase cancer risk. Understanding such non-targeted mechanisms readily lead to potential mechanisms for clinical interventions for health risks in future populations.

## Modeling Radiation Carcinogenesis

Most models of cancer risk and mitigation are focused on “targets,” i.e., the cells that will undergo neoplastic transformation or the genetic alterations that initiate and promote this event. This is classically modeled in which carcinogenesis is thought to occur in four interdependent stages. The first stage is *initiation* and is typically caused by chemical, physical, or biological agents, which irreversibly and heritably alter the cell genome resulting in an enhanced growth potential. This potential is only realized, however, if the cell later undergoes *promotion*, the second stage of carcinogenesis. *Promotion* is often thought to be the rate-limiting step in carcinogenesis since it has been shown that initiation alone is not sufficient to induce cancer (40). In order to account for the observed power of age dependence in radiation-induced carcinomas, a multistage theory of carcinogenesis was introduced very early (41, 42). However, this model suggested five to seven rate-limiting stages, in contradiction with biological data. Some approaches addressed this contradiction by introducing the two-stage clonal expansion model, where a cell leads to a tumor by two separate mutations and clonal expansion (43–45). Integration of specific genetic mutations in tumor suppressor genes was originally introduced by Knudson (46). The current paradigm of carcinogenic risk remains heavily focused on predicting mutations of the genome leading to silencing of tumor suppressor genes or activation of oncogenes. However, such models neglect the influence of intercellular and extracellular interactions in the tumor growth and predict a final tumor that is unrealistic in that its cells are clonally identical.

Systems radiation biology seeks to integrate information about changes across time and scale that are determined by experimentation and to interrogate this to identify the critical events. By modeling the irradiated tissue/organ/organism as a system rather than a collection of non-interacting or minimally interacting cells, cancer can result as an emergent phenomenon of a perturbed system (47). A biological model in which radiation risk is the sum of dynamic and interacting processes could provide the impetus to reassess assumptions about radiation health effects in a healthy population and spur new approaches to prevent detrimental processes that lead to pathology.

Our studies have addressed this problem by separating RTE from NTE by using the mammary gland as a model system. The mouse mammary gland provides an experimentally malleable framework for separating the contribution of NTE on the host from the target epithelium. Mammary gland develops during the postnatal period such that the epithelium can be surgically removed and replaced, creating a tissue chimera. Transplanted syngeneic epithelium can have a specific germ line manipulation, such as a transgene or knockout, or can have received a specific type of exposure, such as radiation. We transplant *unirradiated Trp53 null* mouse mammary tissue into *irradiated* syngeneic wild-type hosts to study whether radiation NTE acting *via* the host affects the process of epithelial carcinogenesis. The *p53 null* mammary model originally described by Medina and colleagues has important features in common with human breast cancer (48). Although about a quarter of human breast cancers have p53 mutations, the utility of this model is that *Trp53 null* mouse mammary tissue develops normally until about 8 months of age, when both ductal carcinoma *in situ* and aneuploidy are evident, thus reproducing the long latency and early instability observed in most human breast cancers. Importantly, the *p53 null* tissue gives rise to histologically heterogeneous tumors that can be estrogen receptor negative or positive and genomically diverse, as are human breast cancers. Thus, the model of an oncogenically primed epithelium lacking p53 condenses the time necessary for spontaneous mutagenic events to accumulate.

The radiation-genetic chimera is used to determine whether and how radiation NTE contribute to mammary carcinogenesis (49). These data from provide strong support that NTE do contribute to radiation carcinogenesis and offer new insight into radiation quality effects that promote aggressive tumors, particularly upon exposure in middle age. Our studies summarized here have identified NTE-mediated mechanisms that include stem cell regulation, inflammation, and immune suppression that are important in determining the rate at which cancers develop and the type of cancer depends on radiation quality and genetic background.

The radiation chimera shows that NTE act *via* the microenvironment to accelerate tumorigenesis and affect critical characteristics (49). A notable observation was that the frequency of ER-negative tumors significantly doubled in irradiated hosts, which was replicated with HZE particle irradiation (50). Importantly, early radiation exposure increased ER-negative tumors in women treated with radiation for childhood cancer fourfold compared to a consecutive series of breast cancers not preceded by radiation (51). A new study by Horst and colleagues confirmed that radiation-preceded breast cancer in survivors of childhood cancer is significantly more likely to the aggressive, the so-called triple negative (negative for ER, progesterone receptor, and amplification of HER2) breast cancer (52). Interestingly, there is little evidence that the frequency of contralateral ER-negative breast cancer is increased in women treated with radiation for breast cancer (53), suggesting a physiological basis for the shift to ER-negative tumors, which are clinically less responsive and more likely to metastasize soon after detection.

To further explore how tumors arising in irradiated hosts are distinct from those that occur in non-irradiated hosts, we profiled total RNA from mammary cancers that arose in non-irradiated mice and irradiated mice (49). Permutation analysis was used to identify 156 genes that segregated tumors from irradiated or non-irradiated hosts. Significant enrichment of genes-involving leukocyte chemo-attraction and binding, monocyte maturation, and proliferation of tumor cell lines underscores the parallels between tumors forming in irradiated host and expression programs activated shortly after radiation exposure, even though the exposure occurred months before and the tumors arose from *unirradiated* epithelium.

We then used this strategy to generate a list of 323 genes and an irradiated host metaprofile (54). Bioinformatics analysis of the human orthologs of the host irradiation metaprofile was used to conduct unsupervised hierarchical clustering of radiation-associated human cancer (54). The irradiated host metaprofile segregated sporadic cancers from radiation-preceded sarcomas (55) and radiation-preceded papillary thyroid carcinomas (56). These analyses support our hypothesis that the microenvironment mediates the development of radiation-preceded human cancers.

Four gene networks representing two cell types, stem cells and macrophages, and two processes, motility and autophagy, were identified in the irradiated host tumor signature. Tissue-specific stem cells or early progenitor cells are considered to be the critical cellular target in carcinogenesis (57–63), based, in part, on the idea that stem cell transformation can lead to unlimited progeny. A mammary stem cell (MaSC) signature, defined by Visvader and colleagues (64), is enriched in the mammary gland up to 1 month after mice are exposed to 10-cGy γ-radiation. We showed this signature is functional as indicated by a doubling of mammary repopulation capacity as well as the pool of cells defined by cell surface markers as associated with mammary repopulation (49). Additional experiments in conjunction with computational modeling led us to conclude that radiation elicits a durable but transient stem cell expansion in a TGFβ and Notch-dependent fashion in juveniles, but not adults (50). In model systems, we found that TGFβ increases self-renewal is blocked by γ-secretase inhibition, indicative of concomitant Notch signaling, which is also induced by low-dose irradiation. This temporary increase in self-renewal is similar to our earlier studies showing that both high- and low LET radiation exposure primes non-malignant human epithelial cells to undergo TGFβ-dependent epithelial–mesenchymal tran­sition (65–67). These studies underscore that even a single radiation exposure can cause phenotypic re-programing.

## Cancer and Inflammation

The concept that inflammatory responses are necessary components of cancer development has recently been formalized by Mantovani et al. (68) in a two-pathway model: the intrinsic versus extrinsic. In the intrinsic pathway, genetic mutations lead to release by the transformed cells of proinflammatory factors recruiting innate immune cells. For example, oncogenic *Ras* activates the transcription of the inflammatory cytokine interleukin-8 (IL-8). Other oncogenes, such as *Bcl2*, inhibit apoptosis leading to necrotic tumor cell death and release of damage-associated molecular pattern molecules that activate innate immune cells *via* toll-like receptors (68, 69). In both circumstances, the resulting host response is a smoldering inflammation that promotes tumor growth and invasion (68, 70). In the extrinsic pathway, the chronic inflammation results from inability of the immune system to resolve an infection (e.g., hepatitis B) or from a dysregulated immune response as in autoimmune diseases (e.g., inflammatory bowel disease). The persistent inflammation cooperates with preexisting oncogenic mutations by providing the microenvironment that promotes cancer progression, but it may also induce DNA damage resulting in the acquisition of new mutations (71, 72).

The innate immune system functions as an “interpreter” of tissue damage that not only provides a first line of defense but also translates the information to wound repair and defense systems in the body by stimulating angiogenesis and activating adaptive immunity. Therefore, it is not surprising that various types of innate immune cells have been found as part of the tumor inflammatory infiltrate. Macrophages play a central role in most solid malignancies, and most studies have found that macrophage abundance, increased microvessel density, and reduced patient survival are highly correlated (73). In fact, macrophages present within tumors are defined as tumor-associated macrophages to denote a specific phenotype that is associated with the production of several proangiogenic factors and cytokines that suppress antitumor immune responses and promote tumor growth by maintaining protumorigenic inflammation.

The application of systems biology by Balmain and colleagues uncovered a differential hub for inflammation in skin cancer (74). While a positive association exists between chronic inflammation and cancer, the innate immune system is itself a network that can be disrupted by both positive and negative stimuli. Anti-inflammatory drugs can have contradictory effects on skin tumor development (75, 76), and over-expression of proinflammatory cytokines, such as IL-1, can prevent skin tumor formation in mouse models of chemically induced skin cancer (77). In contrast, germline deletion of TNF-α, another potent proinflammatory cytokine, also confers resistance to skin tumor formation (78). The role of inflammation in cancer is, therefore, very complex, with different consequences associated with acute or chronic inflammatory conditions.

How the interplay between inflammatory cells and genetically mutated neoplastic cells promotes cancer development and progression remains a subject of intense investigation. Several important pathways have been identified. Among them, IL-6 signaling pathways play a major role (79). Macrophages are the main source of IL-6 during acute inflammation and T cells during chronic inflammation. Importantly, IL-6 orchestrates the transition from acute inflammation, dominated by granulocytes, to chronic inflammation, dominated by monocytes/macrophages and regulates, together with TGFβ, the differentiation of naïve T cells to Th17 proinflammatory phenotype, thus influencing the type of adaptive immune response (80).

Seminal studies by Wright and colleagues identified non-clonal radiation-induced genomic instability in hematopoietic stem cells [reviewed in Ref. (6)], which they now explain as a result of altered cell interactions. Macrophages from irradiated mice could induce chromosomal instability in non-irradiated hematopoietic cells *via* production of TNFα and reactive oxygen and nitrogen species (81). Further studies showed that this effect was a function of mouse genotype, which affects the steady state M1 or M2 macrophage phenotype, which radiation exposure further amplifies (82). HZE particle NTE on inflammatory processes is supported by studies from Burns and colleagues who showed that chronic dietary exposure to vitamin A acetate can prevent almost all malignant and benign tumors that occur in rat skin exposed to electron radiation and most of those following ^56^Fe ion irradiation (83). Gene expression analysis suggested that vitamin A reduced or blocked ^56^Fe ion radiation-induced inflammation-related genes that were represented in the categories of “immune response,” “response to stress,” “signal transduction,” and “response to biotic stress” (84).

To investigate systemic effects of HZE, the *Trp53* null mammary radiation-chimera model was irradiated with low fluences (equivalent to average dose of 11, 30, and 81 cGy) of 350 MeV/amu ^26^Si particles and compared to contemporaneous γ-irradiated (100 cGy) and sham-irradiated mice (85). The median time to tumor detection in mice irradiated with the lowest ^26^Si fluence or γ-radiation was similar to that in sham-irradiated mice but decreased for transplants in mice exposed to higher fluences of ^26^Si particles. As previously reported, the growth rate of tumors arising in irradiated mice was increased compared to those arising in sham-irradiated mice but was significantly faster than high fluence Si-irradiated mice compared to γ-irradiated mice. Since the initial growth rate of tumors arising in hosts irradiated with 11-cGy ^26^Si particles was comparable to that of tumors arising in mice irradiated with 100 cGy sparsely ionizing γ-rays, we concluded that there is an RBE of about 10 for this endpoint.

The carcinoma spectrum arising in mice exposed to ^26^Si particles is enriched for a subclass that is ER-negative and keratin 18-positive. These tumors in Si-irradiated mice developed metastases twice as often as non-irradiated mice. As ^26^Si irradiation of hosts primarily promotes specific ER-negative subtypes, genomic analysis of these tumors compared to a comparable group from sham-irradiated mice. Consistent with these differences, an expression profile that distinguished K18 tumors arising in ^26^Si-irradiated compared sham-irradiated mice was enriched in MaSC, stroma, and Notch signaling genes. These data suggest that the carcinogenic effects of NTE from densely ionizing radiation compared to sparsely ionizing radiation elicit more aggressive tumors. In humans, the type, the density, and the location of immune cells within the tumor are strongly associated with prognosis (86). Together, these data support the hypothesis that radiogenic cancer risk is augmented by alterations in a network of cellular interactions, at the center of which is the innate immune system.

## Immune Surveillance and Suppression

A fundamental role of the immune system is enforcing tissue homeostasis, a task accomplished by mounting inflammatory reactions that involve the coordinated activation of innate and adaptive immune cells. Radiation perturbs tissue homeostasis by activating inflammatory reactions that often do not resolve, leading to a vicious cycle of subclinical tissue damage and smoldering inflammation (87, 88). Whereas one body of work has clearly established the capacity of chronic inflammation to initiate and promote cancer (88), other studies have revealed that an intact immune system can prevent/control and shape cancer by a process best conceptualized in the “cancer immunoediting” theory (89). During initial clonal expansion, recognition of the stressed transformed cells by innate immune cells results in production of interferon-γ, a cytokine shown to play a key role in immunosurveillance against tumors (90, 91). Killing of the preneoplastic cells by natural killer cells or macrophages activated by IFN-γ to produce cytocidal reactive oxygen and nitrogen species eventually leads to cross-presentation by dendritic cells of antigens from the dying tumor cells to T cells and activation of the adaptive immune system. The tumor-specific T cells may be able to destroy completely the incipient tumor, thus functioning as an extrinsic tumor suppressor mechanism that reduces the incidence of spontaneous and carcinogen-induced tumors, something for which there is unequivocal evidence in experimental models and supportive evidence in humans [reviewed in Ref. (39, 92)].

However, if complete elimination of transformed cells is not achieved, the immunological pressure results in selection of clones of cells that have acquired mutations or epigenetic changes conferring resistance to immune rejection, i.e., are “edited” by the immune system to select for those that are poorly immunogenic. This transition from elimination to escape can occur directly or even after a very long period of equilibrium, during which the immune response actively limits progression. The concept of equilibrium, which was initially formulated to explain clinical observations of occult tumors and tumor dormancy (93, 94), has been confirmed in experimental models showing that depletion of T cells leads to growth of occult tumors (95). Importantly, protumorigenic inflammation and antitumor immunity can co-exist in the same tumor, and interventions that can alter the balance in favor of one or the other may either accelerate or hinder tumor growth (88).

We found that lymphocyte infiltrate of *Trp53* null tumors arising in the irradiated mammary chimera correlates to tumor growth rate, i.e., faster growing tumors have less lymphocytic infiltrate, and that particle irradiation elicits the most rapidly growing tumors. This observation suggests that HZE particles have a systemic impact on the immune surveillance that leads to the development of more aggressive tumors.

## Genetic Mediators of Cancer

Epidemiological and genetic studies show that there is a strong genetic component that contributes to the differences between individuals in their response to DNA damage and cancer susceptibility (96, 97). High penetrance mutations in genes, such as BRCA1/2, are responsible for a proportion of cancers that show familial aggregation (98). However, the genetic basis of susceptibility to the majority of cancers that have no obvious familial aggregation is almost completely unknown (96, 99). Most studies to identify susceptibility loci for radiation-associated cancer are limited to candidate genes involved in response to DNA damage, but there is strong evidence that other processes are important; systems genetics seeks to uncover those components that result from complex interactions between pathways and cells.

Systems genetics, unlike traditional approaches to the analysis of disease that focus on single genes or proteins in isolation, attempts to integrate the complex interaction of many kinds of genetic and biological information – genomic DNA sequence, mRNA, and protein expression, and link these to disease phenotypes. Human studies have demonstrated strong associations between polymorphic variation and regulation of gene expression (100–102). Parallel studies in mice offer many advantages for the study of the genetic basis of complex traits. The ability to control genetic background and to carry out crosses between mouse strains differing in their propensity to develop these diseases offers unprecedented opportunities to identify and investigate the primary genetic loci that control susceptibility. In addition, studies with mice allow precise exposures, standardized husbandry to control other environmental components of risk, and comprehensive analysis of phenotypes.

Applying these approaches to mouse strains with differing susceptibility to diseases identifies signaling hubs that may be important targets for therapy or prevention (103). A systems genetics approach consists of a network view of the genetic and gene expression architecture of normal host tissues that are compared after perturbation by radiation or tumor development (104, 105). An example of this strategy used gene expression profiles of skin from a population of *mus spretus* backcrossed to *mus musculus* mice to reveal the normal skin gene expression motifs associated with sensitivity to carcinogen-induced skin tumor development in contrast to those that were resistant. This analysis revealed both cell-autonomous (cell cycle and stem cell lineage) and non-cell-autonomous (inflammation and innate immunity) components that were differentially expressed in the susceptible animals. Interestingly, the highly susceptible mice exhibited increased levels of anti-inflammatory genes within the inflammation associated network, leading to the conclusion that chronic and acute inflammation are, respectively, tumor-promoting versus suppressive (106).

Multiple tumor types in mice, including thymomas, soft tissue sarcomas, and osteosarcomas, can be induced by exposure to low LET radiation, but induction is typically infrequent and tumors have long latency (i.e., survival time post-radiation). Engineered loss or misregulation of p53 increases the detection sensitivity. Radiation induces the same spectrum of tumors in p53-deficient mice that lack one or both p53 alleles; however, the survival time is dramatically reduced after a single exposure to ionizing radiation (107). Likewise, the *Trp53* null BALB/c inbred mouse strain is sensitive to mammary carcinogenesis, and radiation exposure enhances this susceptibility (108–110). The utility of this model is that tumors are diverse by all criteria, markers, histology, metastatic capacity, and genomic profiling, in a fashion that is remarkably aligned with human breast cancer (48, 111).

Recent experiments focus on the genetic contribution to NTE using the mammary chimera (112). Radioresistant SPRET/EiJ was mated to radiosensitive (BALB/c) mice, and then the progeny were backcrossed to BALB/c to generate F1 backcrossed mice (F1Bx). Our prior experiments using inbred BALB/c mice showed that host irradiation decreased *Trp53* null tumor latency, increased frequency of tumor formation at a year posttransplantation, and that tumors arising in irradiated hosts grew more rapidly (49). Consistent with our previous observations, the growth rate of *Trp53* null mammary carcinomas was greater in irradiated F1Bx host mice, a feature associated with aggressive tumors, compared to unirradiated mice. However, *Trp53* null tumor latency increased in irradiated hosts and tumor frequency was reduced by 9.6% (*p* = 0.04) at 18 months posttransplantation compared to sham-irradiated F1Bx hosts. The revelation that NTE delay rather than accelerate mammary cancers in genetically diverse hosts underscores the outcome of radiation exposure in terms of carcinogenesis depends of genetic background.

Introgression was used to determine the genetic loci that affected *Trp53* null mammary tumor latency of the radioresistant SPRET/EiJ genome using genome-wide genotyping. Only two loci were associated with tumor latency in sham-irradiated mice. Tumors in mice homozygous for the BALB/c allele at loci on chromosomes 2 and 14 appeared with a significantly shorter latency than those mice, in which one allele was from BALB/c and the other from SPRET/EiJ at these loci. Interestingly, neither of the loci affected latency in irradiated hosts. In contrast, 15 genetic loci were associated with tumor latency in irradiated mice, 11 alleles confer resistance to tumor development, and 4 alleles conferred susceptibility.

Together, the use of systems genetics with the radiation-chimera model provides new insight into the processes that mediate carcinogenic susceptibility to radiation. To further explore stromal genetic associations with cancer risk after exposure to low LET radiation, we used ingenuity pathway analysis (IPA) to identify 696 candidate genes located within the identified loci. Of these, 185 genes were within 4 loci on chromosomes 2, 11, 14, and 16 where homozygous BALB/C alleles associate with increased latency for cancer arising in irradiated mice. These genes were enriched in four pathways, γ-glutamyl cycle, leukotriene biosynthesis, alanine biosynthesis III, and glutathione biosynthesis. In contrast, 511 genes enriched for 24 pathways were within 11 regions where heterozygous SPRET/EiJ alleles associate with increased latency. Importantly, these 11 loci were enriched for genes involved in regulating the immune response including signaling pathways of natural killer cells and cytokines. Radiation-induced activation of pathways that control release of inflammatory cytokines varies among mouse strains (113, 114) and is postulated to contribute to genetic susceptibility to radiation-induced leukemia (113). Analysis of the upstream regulators of these candidate genes indicated that the TGFβ and p53 pathways might also be involved in mammary tumor susceptibility.

The observation that many more genetic loci are linked with tumor latency in the radiation-treated cohort than in the sham-irradiated cohort suggests the interesting idea that genetic contribution is actually specific to NTE, in contrast to the widely held belief that radiation exaggerates inherent susceptibility. This is exemplified by the work of Onel and colleagues who identified PRDM1 (Blimp-1), a transcriptional regulator of cell specification, with the risk of second malignancies only in those treated with radiation for childhood malignancy (115). In individuals with the homozygous protective allele, the incidence of second cancers is 3:100 by 30 years after exposure, whereas in those who were homozygous for the allele, risk is 1:3. Thus, the risk allele conferred risk comparable to BRCA1 mutation, but only in the context of radiation.

## Summary

Identifying mechanisms of NTE is essential to understand the biology of irradiated tissues. Two fundamental aspects of NTE in carcinogenesis warrant careful consideration for further understanding of cancer risk in irradiated populations. First is that radiation NTE may alter the shape of the dose response. Recent modeling by Cucinotta and colleagues suggest that NTE may be particularly important in the low-dose region of concern for occupational exposures. Second, NTE are targetable; the biology that ensues after exposure is persistent and may be “reset” after the fact to limit carcinogenic potential. This offers the possibility of protecting those at greatest risk, for example, children who are treated with charged particles for childhood malignancy, in which the clear benefit of dose distribution may come at the price of long-term cancer risk. Moreover, NTE will likely provide insight into the use of particles for cancer therapy as there are common microenvironment components, such as the immunoregulatory axis and the vasculature, that are likely critical to treatment outcome.

## Author Contributions

Dr. MHB-H outlined and wrote the manuscript. Dr. J-HM edited and contributed to this manuscript.

## Conflict of Interest Statement

The authors declare that the research was conducted in the absence of any commercial or financial relationships that could be construed as a potential conflict of interest.

## Abbreviations

HZE, high *Z* and energy; LET, linear energy transfer; NTE, non-targeted effects; RBE, relative biological effect; RTE, radiation targeted effects; TGFβ, transforming growth factor β.
